# Utilizing Dietary Nutrient Ratios in Nutritional Research: Expanding the Concept of Nutrient Ratios to Macronutrients

**DOI:** 10.3390/nu11020282

**Published:** 2019-01-28

**Authors:** Owen J. Kelly, Jennifer C. Gilman, Jasminka Z. Ilich

**Affiliations:** 1 Abbott Nutrition, 2900 Easton Square Place, 01C13A/ES1, Columbus, OH 43219, USA; jennifer.gilman@abbott.com; 2 Institute for Successful Longevity, Florida State University, Tallahassee, FL 32306, USA; jilichernst@fsu.edu

**Keywords:** dietary guidelines, nutrient ratios, dietary patterns, macronutrients, protein, carbohydrate, fat, dietary reference intakes, daily values

## Abstract

We recently showed that using micronutrient ratios in nutritional research might provide more insights into how diet affects metabolism and health outcomes, based on the notion that nutrients, unlike drugs, are not consumed one at a time and do not target a single metabolic pathway. In this paper, we present a concept of macronutrient ratios, including intra- and inter-macronutrient ratios. Macronutrient intakes from food only, from the What We Eat in America website (summarized National Health and Nutrition Examination Survey data) were transposed into Microsoft Excel to generate ratios. Overall, the dietary ratios of macronutrients may be more revealing and useful in epidemiology and in basic nutritional research than focusing on individual protein, fat, and carbohydrate intakes. While macronutrient ratios may be applied to all types of nutritional research, nutritional epidemiology, and, ultimately, dietary guidelines, the methodology required has not been established yet. In the meantime, intra- and inter-macronutrient ratios may serve as a measure of individual and total macronutrient quality.

## 1. Introduction

It is common and widely accepted to report dietary intake and/or nutritional status relating to some health outcomes relative to single nutrients. We have already introduced the concept of using nutrient ratios in research for the micronutrients [1], as well as presenting the overall rationale for utilizing nutrient ratios, based on the physiological and metabolic properties of nutrients. Our rationale is based on four basic and well-established principles:

1. Meals contain an assortment of nutrients, not a single nutrient.
2. Each meal or food item has a different assortment (ratio) of nutrients.
3. Most of the nutrients function in various tissues and are not tissue specific [2].
4. Nutrients are not limited to one specific metabolic pathway [2].

We have previously reported that, in general, diets are low in dietary fiber, high in simple carbohydrates, and low in eicosapentaenoic acid and docosahexaenoic acid but higher in protein compared with dietary recommendations [3]. However, aggregate measures, such as total dietary protein, fat, and carbohydrate (CHO) do not reveal the quality of macronutrients in a diet. For example, a recent focus on added sugars enhances the need to investigate macronutrient ratios (an example for CHO could be the ratio of simple sugars to complex sugars to added sugars) to determine quality and how specific ratios of macronutrients affect health. This notion applies to other macronutrients as well, such as ratios of saturated to polyunsaturated fatty acids or essential to nonessential amino acids as components of total fat and protein, respectively.

It is possible that evaluating macronutrient ratios may provide a measure of dietary macronutrient quality and how proteins, fats, and carbohydrates, as part of a total diet, affect health outcomes. However, macronutrients differ from micronutrients in several fundamental ways:

- Macronutrients are groups of different compounds, which may have distinct functions, classified by common features.
- Macronutrients are utilized for energy production; the intensity of activities [4] or health condition [5] determine which macronutrients are catabolized.
- Macronutrients are usually considered to be just proteins, carbohydrates, and fats, which only provides the ratio of three values; this may have limited usefulness.
- Unlike vitamins and minerals, macronutrients lack a unified set of dietary intake recommendations apart from the daily values (DV), which are designed for use on food labels [6].

As an example, the limitations of single nutrient studies are highlighted by the Osteoporotic Fractures in Men (MrOS) Research Group, who found that separate macronutrients, at baseline, did not show consistent associations with change in frailty status (defined as robust, intermediate, or frail) at follow-up (mean 4.6 years), whereas a worse overall diet quality was positively associated with frailty at follow-up [7]. This shows that the overall diet (nutrient ratios) is more important than its constituent parts (single nutrients). We have previously published findings from the National Health and Nutrition Examination Survey (NHANES) summary data from What We Eat in America, which show that the distribution of macronutrients in the United States (US) diet may contribute to osteosarcopenic obesity, a condition encompassing the concurrent appearance of osteopenia/osteoporosis, sarcopenia, and obesity [3,8]. Beyond simply focusing on ratios of total protein, fat, or carbohydrate, internal (intra-macronutrient) and external (inter-macronutrient) macronutrient ratios can be considered in nutritional research. Examples of intra-macronutrient ratios would be the n-6/n-3 polyunsaturated fatty acid (PUFA) ratio or ratios of individual amino acids from dietary protein, whereas an example of an inter-macronutrient ratio would be the ratio of the essential fatty acids to added sugars, fiber, tryptophan, and glycine. The most basic inter-macronutrient ratio is the ratio of total protein to total fat and total carbohydrate.

A murine study by Solon-Biet and colleagues [9] showed that modifying the protein/carbohydrate ratio inhibited mammalian target of rapamycin (mTOR) activation, independent of energy intake. A possible mechanism is that the lower dietary protein/CHO ratio resulted in reduced circulating branched-chain amino acids and higher blood glucose, thus reducing mTOR activation, which is linked to better aging [10]. On the other hand, the mice consuming the highest protein/CHO ratio diets had higher mTOR and insulin levels but the lowest food energy intake. Li et al. investigated protein/CHO ratios in lean and obese dogs and found that different protein/CHO ratios have distinct effects, particularly in the microbiome of obese dogs [11]. Chang and Huang investigated the effect of the PUFA + monounsaturated fatty acids (MUFA) to saturated fatty acids (SFA) ratio on plasma and liver cholesterol in rats and found that to maintain a good cholesterol level, the following are important: a low MUFA/SFA, a high PUFA/MUFA ratio, and the ratio of PUFA+MUFA/SFA should be ≤2 [12]. The low MUFA/SFA ratio is counterintuitive to current dietary guidelines for humans; however, Chang and Huang explain that with a fixed PUFA/SFA ratio of 1, high MUFA levels increase liver cholesterol in murine studies; investigating these ratios in human studies may provide some insight into how ratios of particular fatty acids affect the role of other fats.

For humans, consumption of low CHO/high CHO diets in studies are indirectly modifying the inter-macronutrient ratios. While reviewing all these studies is beyond the scope of this concept paper, a reanalysis of these studies may yield some valuable insight into macronutrient ratios. A recent meta-analysis, using US data, found that mortality increased at both low (<40%) and high (>70%) CHO intakes (U-shaped curve) [13]; however, the sources of the other macronutrients are key to the interpretation of this finding, with plant-based protein and fats providing a lower mortality risk at the low CHO level. Approximately 85% of energy intake in the Okinawan diet is from CHO and only 9% of energy from protein [14]. Okinawa residents were among the longest living [15] and consumed a diet with a protein to carbohydrate ratio of 1/10 [14], as opposed to an approximate ratio of 1/3 from the NHANES data [3] and a DV ratio of 1/5 (the DV for protein and carbohydrate are 50 g/day and 275 g/day, respectively). While specific diet quality scores for the Okinawan diet are not available, it is high in fruit and vegetables and low in refined grains, showing that the high carbohydrate intake is predominately unrefined and low glycemic index carbohydrates [16]. Furthermore, the Okinawan diet is lower in energy, higher in n-3 PUFA, and higher in functional phytochemicals [17]. This Okinawan diet supports the U-shaped CHO curve analysis by Seidelmann et al. [13], as intakes of plant-based foods are higher, even though 85% of energy intake is CHO [14], and also suggests that inter- and intra-macronutrient ratios are important determinants of health outcomes. Therefore, intra-macronutrient ratios could help measure individual macronutrient quality, whereas inter-macronutrient ratios could help measure diet quality with any associated health outcomes.

Based on the above, it is reasonable to consider that intra- and inter-macronutrient ratios could be a measure of macronutrient quality, and once defined ratios are established from studies, there may be distinct ratios for each condition or health benefit. Therefore, the overall aims of this paper are to extend the nutrient ratio concept [1] to macronutrients, determine what is possible with the current macronutrient intake data, and identify the gaps in the macronutrient data.

## 2. Materials and Methods: Approach to Analysis and Encountered Limitations

The overall approach has already been described in our previous paper [1]. Briefly, we summarized the National Health and Nutrition Examination Survey (NHANES) 2001–2016 food intake data, published on the What We Eat in America website [18]. The data were transposed into Microsoft Excel. The total mean daily macronutrient intakes, as well as the intakes at eating occasions, were included. During our analysis, we encountered the following limitations. The What We Eat in America data tables did not report individual amino acids, and the CHO details were also limited; however, the total CHO, total sugars, and total fiber were reported. The What We Eat in America tables do contain detailed information on the lipid composition of the diet.

The Health and Medicine Division of the National Academies of Sciences, Engineering, and Medicine have developed the most complete dietary reference intakes (DRIs) for the United States. DRIs exist for total protein, total carbohydrate, fiber, linoleic acid, and α-linolenic acid for people aged 1 year or more [19]. In addition, the DRIs include the Acceptable Macronutrient Distribution Ranges (AMDR). AMDRs exist for protein (10–35% of energy), fat (20–35% of energy), and carbohydrate (45–65% of energy), but because they are ranges, they were difficult to use in ratio calculations and when trying to compare dietary intakes to recommended intake values. Therefore, using the DRIs would have limited the ability to create macronutrient ratios.

A second set of US government recommendations for macronutrients exists, which is based on the DRIs. Food labels report the Daily Value (DV), which is usually represented as both a value (e.g., mg) and a percentage of the DV (%DV), both based on a 2000 kcal dietary intake for ages 4 and above. The overall purpose of the DVs is to aid an individual to assess the nutritional content of a food and even compare foods, label-to-label. The DVs themselves contain two sets of daily nutrient amounts for food labeling purposes: (1) Daily Reference Values [20] and (2) Reference Daily Intakes [21]. DRVs cover fat, saturated fat, cholesterol, carbohydrate, fiber, sodium, potassium, and protein. RDIs cover vitamins and minerals. Both contain values for children less than 4 years of age and for pregnant and lactating women; however, the most complete DVs are for those aged 4 years or older (see Table 1). The Food and Drug Administration revisions for the DVs went into effect in 2016 (81 Fed. Reg. 33742, 27 May 2016), and we utilized these new values. In addition, the DVs do not vary by sex and age and are one value based on a 2000 kcal diet, meaning one value for each nutrient will cover the majority of the population. The DVs contain specific gram quantities for several macronutrients, which made them ideal for comparing intake ratios to standard values. However, the DVs, similar to the DRIs, lack specific recommendations for high and low glycemic index digestible carbohydrate, total simple sugars (mono- and disaccharides but do include added sugars), MUFA, PUFA, and many others (e.g., soluble versus insoluble fiber). The DVs, while appropriate for food labeling purposes, confer very little information about the quality of the macronutrients. However, this issue is not exclusive to the DVs; dietary guidelines do not provide recommendations for all proteins, carbohydrates, and fats. While the DVs may not be considered ideal for research, they are rooted in the DRIs and act as a best fit for the entire population aged 4 years or older. For this reason, as well as the lack of sufficient benchmarks in the dietary guidelines, we chose to use the DVs in our evaluation of intra- and inter-macronutrient ratios. Therefore, in our analysis, the total and individual macronutrient ratio values from food only were compared with the DVs.

While the DVs do not integrate essential fatty acids into the value for total fat, there is a DV for SFA. There are DRIs for essential fatty acids, but we did not want to mix the DRIs and DVs. There are DVs for added sugars and fiber but no DV (or DRI) for total sugars. However, the values for added sugars were not contained within the What We Eat in America data tables. The DV for saturated fatty acid was subtracted from the DV for total fat with the remainder referred to as “other fat”. The DV for fiber was subtracted from the total carbohydrate to provide “net CHO”. Therefore, the following five-compartment macronutrient model emerged from the DVs:

1. Protein;
2. Saturated fatty acid (SFA);
3. Other fat (total fat minus SFA);
4. Net carbohydrate (net CHO; total carbohydrate minus fiber); and
5. Fiber.

Total macronutrient intakes and recommendations were calculated by adding together values for protein, fat, and carbohydrate. To calculate the ratios, the intake or DV value was divided by the sum of all the intake values for macronutrients in the set:

- Ratio of protein = protein/(protein + SFA + other fat + net CHO + fiber);
- Ratio of SFA = SFA/(protein + SFA + other fat + net CHO + fiber)
- Ratio of other fat = other fat/(protein + SFA + other fat + net CHO + fiber)
- Ratio of net CHO = net CHO/(protein + SFA + other fat + net CHO + fiber)
- Ratio of fiber = fiber/(protein + SFA + other fat + net CHO + fiber)

For the calculations check, the sum of all the ratios must be = 1.

As we showed with the micronutrient ratios concept [1], an alternative way to express the macronutrient ratios is to express them relative to another macronutrient. We chose protein for the inter-macronutrient ratios, as it is probably the most studied macronutrient [24] and was recognized as the first nutrient [25]. Detailed intake data were available for fat from the What We Eat in America data tables, so intra-macronutrient ratios are presented relative to oleic acid n-3 (MUFA 18:1), as we found it was the fatty acid, which was consumed in the highest quantity by the NHANES participants. Intra-macronutrient ratios for protein were not possible, and the CHO intra-macronutrient ratios were limited to sugars/fiber/remaining CHO. To calculate the ratio relative to protein, the ratio value for each macronutrient was multiplied by the reciprocal of the protein ratio value (the same method was used for the fatty acid ratios relative to oleic acid):

- Ratio of protein × (1/ratio of protein) = 1
- Ratio of SFA × (1/ratio of protein)
- Ratio of other fat × (1/ratio of protein)
- Ratio of net CHO × (1/ratio of protein)
- Ratio of fiber × (1/ratio of protein)

To limit the number of figures and tables, the male and female 50–59-year-old age groups from the 2007–2008 and 2015–2016 NHANES surveys were used as examples. The 2007–2008 survey year was used in our previous micronutrient ratio paper [1], so it was also used here. The 2015–2016 surveys contain the most recent dietary intake data for the US population.

## 3. Results

### 3.1. Total Macronutrient Intakes from Food

**Figure 1:**
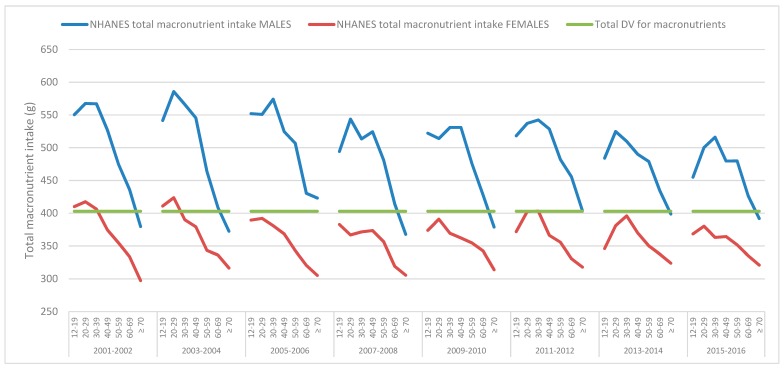
Mean daily total macronutrient intakes, from food, derived from the National Health and Nutrition Examination Survey (NHANES) 2001–2016 data, across various age groups for males and females and the total macronutrient suggested by the Daily Values (DV).

Figure 1 suggests that in general (with some exceptions), males consume more grams of total macronutrients, whereas females consume less than the DVs. The overall pattern is for total macronutrient intake to fall with age in males and females, accompanied by an overall reduction in energy intake with age, as we previously reported [3].

### 3.2. Protein, Fat, and Carbohydrate

**Figure 2:**
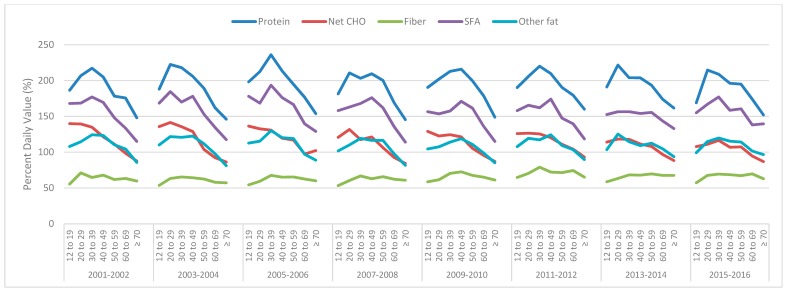
Mean daily male total protein, net carbohydrate (CHO), fiber, saturated fatty acids (SFA), and other fat intakes derived from the National Health and Nutrition Examination Survey (NHANES) 2001–2016 data across various age groups as a percentage of the daily value (100% = daily value).

**Figure 3:**
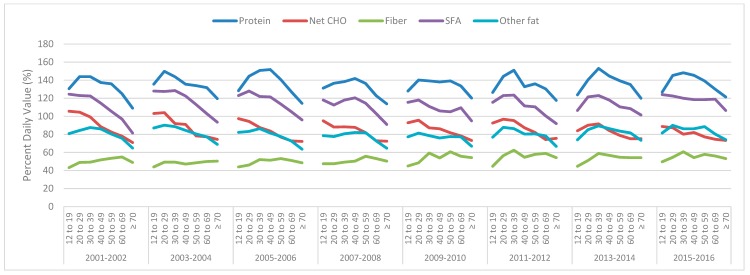
Mean daily female total protein, net carbohydrate (CHO), fiber, saturated fatty acids (SFA) and other fat intakes derived from National Health and Nutrition Examination Survey (NHANES) 2001–2016 data, across various age groups, as a percentage of the Daily Value (100% = Daily Value).

The mean protein intake remained above the DV for all the survey years and age groups for males (Figure 2) and females (Figure 3). Males over 70 years of age for all the survey cycles (and in some survey cycles, for those 60–69 years of age) did not meet the DV for other fat (Figure 2), while females of all ages and survey cycles were below the DV (Figure 3). Males and females of all ages and in all the survey cycles were above the DV for SFA (Figure 2 and Figure 3). Males aged 60+ were below the DV for net CHO, except for in the 2005–2006 survey (Figure 2). Female carbohydrate intakes are more interesting. From the 2005–2006 survey cycle onwards, females of all ages did not meet the DV, while in the previous two survey cycles, only females 12–29 years of age were just above the DV (Figure 3). Males or females never reached the DV for fiber in any survey year or age group.

### 3.3. Mean Daily Macronutrient Intakes from Food at Meal Occasions

For these data, only male and female data from 2007–2008 and 2015–2016 were used to limit the tables and figures. Meals were defined and reported by NHANES participants. Supplementary Figures S1–S6 show that dinner remains the largest meal of the day for males and females of all ages and has the highest energy, protein, fiber, SFA, and other fat. Lunch and snacks have similar total macronutrient intakes for males and females across ages; however, lunch is higher in protein, fiber, SFA, and other fat but lower in Net CHO. Breakfast has the smallest total macronutrient intake, but breakfast is higher in protein, lower in fiber, and has a similar SFA intake compared to snacks for the 2015–2016 data but is lower in SFA for the 2007–2008 data and lower in net CHO and lower in other fat compared with snacks for females but not males.

### 3.4. Macronutrient Ratios

The DV ratios differ from the NHANES intakes from food. Table 2 shows the ratios of macronutrients for the DVs and NHANES data for males and females 50–59 years of age from the 2007–2008 and 2015–2016 survey cycles. 

As males consume more total fat than females, the intakes of individual fatty acids are also lower, in most cases for females (Supplemental Figure S7). The 2015–2016 data suggest that the eight major fatty acids in the US diet from food in males and females are oleic acid (MUFA 18:1), linoleic acid (PUFA 18:2), palmitic acid (SFA 16:0), stearic acid (SFA 18:0), myristic acid (SFA 14:0), α-linolenic acid (PUFA 18:3), palmitoleic acid (MUFA 16:1), and lauric acid (SFA 12:0), with all the remaining fatty acids accounting for approximately 3% of the total fat intake. The three highest consumed SFA, palmitic acid (SFA 16:0), stearic acid (SFA 18:0), and myristic acid (SFA 14:0), account for 90% of the SFA with stearic acid (SFA 18:0) accounting for 56% of the total SFA, showing that US diets are relatively low in shorter chain SFA. MUFA accounts for 39% of the total fatty acid intakes in both males and females. The predominant MUFA is oleic acid (MUFA 18:1) at 95% of the total, followed by palmitoleic acid (MUFA 16:1) at 4% of the total with the remaining two MUFA accounting for the remainder in both males and females, although the total MUFA intake was lower in females. The total PUFA was 26% of the total fatty acids in males and 27% of the fatty acids in females. Linoleic acid (PUFA 18:2) is by far the most abundant PUFA in US diets, accounting for 89% of the total PUFA, and α-linolenic acid (PUFA 18:3) is 10% of the total PUFA, with the remainder (1%) being all the other longer chain PUFA. Table 3 shows the intakes from the food, ratios, and ratios relative to oleic acid (MUFA 18:1) from the NHANES survey years 2007–2008 and 2015–2016 for males and females 50–59 years of age. The data for 2015–2016 were also plotted as pie charts, see Supplemental Figures S8 and S9.

The primary meal macronutrient differences were in the grams of net CHO per gram of protein; all the meals were low in fiber. Snacks had the highest grams of net CHO per gram of protein, followed breakfast and dinner (see Table 4).

## 4. Discussion

This is the first time, to our knowledge, that nutrient intakes were compared to DVs. The total daily macronutrient intakes fall sharply as age increases; this pattern is common to males and females across all survey years and reflects the drop in energy intake with age [3]. However, we observed that females of all ages after 2004 did not meet the total macronutrient intake for the DVs (403 g). A decline in macronutrient intake was also observed in males but only dropped below the DV in those over 70 years of age (Supplemental Figures S10 and S11). A possible explanation may be that the higher protein intake is lowering the intake of other macronutrients (Supplemental Figures S10 and S11); however, the issue may be that the DV for protein is too low. Raising the protein recommendation for adults has been addressed by other researchers [26,27]. The DV for protein (50 g/day) equates to 0.7 g/kg/day for a 70 kg (154 lb.) person, which is below the individual protein recommendation of 0.8 g/kg/day (56 g/day for a 70 kg person [19]), especially considering that the average body weights in the US are 89 kg (195.7 lb.) and 77 kg (168.5 lb.) for males and females, respectively [28].

At the population and individual level, examining single macronutrient (protein, carbohydrate, and fat) intakes may be too ambiguous considering what is expected from nutritional science. Nutritional concerns have shifted from overt malnutrition or nutrient deficiencies (e.g., kwashiorkor)—although it is still present in some areas—to elucidating the effects of energy dense, nutrient insufficient diets [1]. As previously mentioned, the U-shaped curve relationship between the percentage of energy from carbohydrate and all-cause mortality suggested by Seidelmann et al. [13], with the lowest risk at 50–55% is noteworthy, considering the expected differences in dietary CHO quality in the study population. The importance of CHO quality is supported by the finding that the mortality risk changed depending on the source of the macronutrients, with plant-based foods reducing the risk [13]. The NHANES data used here show that sugars made up approximately 40% of the total CHO and fiber only made up approximately 5% of the total CHO, suggesting poor CHO quality intake. However, it would be anticipated, based on the dietary guidelines, that at higher CHO intakes (>55% of energy) all-cause mortality [13] would not be affected if the CHO were high quality (whole grain, low glycemic index, and high fiber). This suggests that intra-macronutrient CHO ratios, and possibly inter-macronutrient ratios, may help explain the study results.

There are some data to support the amino acid ratios (intra-macronutrient protein ratios), as the requirements have been somewhat established for the essential amino acids (not all 20 amino acids) in some age groups [29]. However, the amino acid requirements for sex and chronic conditions are unknown. Further, there is an argument to support only focusing on the essential amino acids, but there are situations where nonessential amino acids become conditionally essential. Philips et al. suggested that each amino acid be treated as an individual nutrient [30], and doing so would provide the ability create complete intra-macronutrient protein ratios. Learning how ratios of amino acids behave in a multitude of situations would help build better dietary guidelines. Protein deserves more attention to elucidate the mechanisms of action of intra- and inter-macronutrient ratios of amino acids and their metabolic, health, and clinical outcomes.

The 2015 Dietary Guidelines for Americans [31] reflect the importance of the quality of fat over quantity, although the same restriction for SFA (<10% of total energy) remains. This is in response to data that question the validity of minimizing fat and SFA intakes in relation to cardiovascular disease, for example [32,33]. There are no reference intakes for individual fatty acids apart from the essential fatty acids linoleic acid (PUFA 18:2) and α-linolenic acid (PUFA 18:3), and the data suggest the US diet is already sufficient with respect to these fatty acids [3]. Hu and colleagues found it difficult to separate out the effects of individual SFA, especially stearic acid [34], and the issue for Hu et al. was that the SFA are all from the same food groups. Palmitic acid and stearic acid contributed, on average, 55% and 16% of the total SFA intakes, respectively; the major food sources were beef and cheese. The data for specific SFA are limited primarily to cardiovascular-related outcomes (which may be linked to higher CHO intake within the context of high SFA) [35], but other outcomes may also be important; for instance, males consuming higher levels of palmitic acid and stearic acid were at an increased risk for gallstone disease [36]. Coconut oil is predominately (~90%) SFA [37], and while its health benefits are promoted, the data are not yet sufficient to exclude short- and medium-chain SFA from dietary recommendations [38]. Interestingly, higher levels of very long chain saturated fatty acids: arachidic acid (C20:0), behenic acid (C22:0), and lignoceric acid (C24:0), in plasma and erythrocytes, were each associated with better blood lipid profiles and lower insulin resistance and inflammation, and a reduced overall risk of coronary heart disease [39].

The abundance of data on fat intake from foods allowed for the intra-macronutrient fat ratios to be determined. It is possible that the current intakes of MUFA and PUFA are not optimal in relation to SFA; people are not consuming SFA alone. There is already some animal evidence that supports the importance of certain intra-macronutrient fat ratios. Chang and Huang suggest that, to maintain blood and liver cholesterol in a healthy range, a low MUFA/SFA ratio (~1), high PUFA/MUFA ratio (~2), and a PUFA+MUFA/SFA ratio not to exceed 2 are required [12]. Legrand et al. show that with a LA/ALA ratio of 4, short-chain SFA may lower blood cholesterol and increase tissue PUFA [40]. The data here for males and females aged 50–59 years of age from the NHANES 2015–2016 show a MUFA/SFA ratio of 1, a PUFA/MUFA ratio of 0.7, a PUFA+MUFA/SFA ratio of 2, and a LA/ALA ratio of 9, suggesting that the negative effects of SFA in the US diet may be due to an unbalanced intake of other fatty acids. This implies that there may not be enough of a variety of fatty acids in our current diet.

The current approach to CHO recommendations focuses on total CHO, fiber, and added sugars; however, there are more specific types of CHO, and they are underutilized. Added sugars are currently an important topic in nutrition; however, the What We Eat in America summary tables did not include added sugars. Therefore, values for sugars, fiber, and total carbohydrate were used to calculate a simple intra-macronutrient carbohydrate ratio, see Table 5. Sugars and fiber were added, and this number was subtracted from total carbohydrates to provide the ‘remaining CHO’. For both the NHANES 2007–2008 and 2015–2016 data, males and females of 50–59 years of age, consumed 7% and 8% of carbohydrates as fiber, respectively, showing fiber intakes have not improved over time. Sugars and remaining CHO percentages were similar, showing that sugars are predominant in the US diet. Between 2007 and 2016 there was a decrease of 1 g of remaining CHO for every gram of fiber consumed, while 7 g of sugars for every gram of fiber did not change for males (Table 5). This suggests that males may be attempting to reduce CHO intake, while females reduced their intake of sugars from 7 g to 6 g per gram of fiber between 2007 and 2016, indicating better CHO choices (Table 5).

Sugars are a major contributor to the CHO of US diets, yet the sugars are not identified, and each specific sugar’s intake is not measured. Fructose consumption was estimated from NHANES III (1988–1994) and ranged from 73 g/day (12% of energy) for 12–18-year-olds to 38 g/day (9% of energy) for those over 70 years of age [41]. Nevertheless, an analysis of fructose intakes by Marriott and colleagues proposed that the relatively small rise in fructose intakes from 1977–2004 were minimal compared to the increases in total energy and CHO [42]. Even a 75 g bolus of glucose was shown to have an inflammatory effect over 3 h, compared with saccharine [43]. In studies, negative health consequences may not be associated with fructose at ≤50 g/day (or ~10% of total energy) [44], and sucrose at >25% of total energy [45], yet participants in these studies may have been consuming other types of sugars, and other CHO, especially low fiber, which may have had a positive effect on outcomes. While diets with combinations of sugars (intra-macronutrient CHO ratios), such as with 10% of energy from fructose and 25% from sucrose, have not been studied, a reanalysis of previous studies where specific sugars were increased or decreased may yield to important data on intra-macronutrient CHO ratios and health outcomes. The routine reporting and analysis of more detailed protein, fat, and CHO may help clarify the roles of intra- and inter-macronutrient ratio intakes in health.

We have already introduced the concept of proportionality in our micronutrient ratio concept paper [1]. Using the 2007–2008 and 2015–2016 NHANES summary data (from What We Eat in America) for males and females aged 50–59 years, we recalculated the ratios for macronutrients as if they were consumed in the same proportions defined in the DVs (i.e., what would intakes look like if the NHANES participants followed the DV ratios). To calculate proportions, the total macronutrient intakes were multiplied by the DV ratio (equivalent to the percent of total intake). Table 6 shows that in relation to the DVs, males and females (aged 50–59 years) consumed more protein, too few CHO and fiber, and more saturated fat based on their respective total daily macronutrient intakes. While the macronutrient distribution for the NHANES intakes in proportion to the DVs are within the AMDR, the intake of protein and fat look very different. Proportionality may be more relevant once more detailed amino acid and CHO data are available.

This paper expands the concept of utilizing nutrient ratios to macronutrients. Nutritional needs in the 21st century have changed, and a systems approach to diet and health is required, especially as nutrition is shifting towards dietary patterns. There is a need to measure and report more detailed macronutrient composition information, as total protein, fat, and carbohydrate convey very limited information, especially when correlating these values with health outcomes. Analyzing detailed dietary intra-macronutrient ratios may help establish better macronutrient quality benchmarks, and in combination with inter-macronutrient ratios, they may help shape future guidelines to prevent and treat modern disease and conditions.

## 5. Conclusions

Based on the presented analyses and discussion, it is obvious that knowing just the dietary intakes for total protein, fat, and carbohydrate do not reflect any information about the quality of macronutrient intakes, and there is a need to know more about the composition of the modern diet and how it shapes modern diseases.

Nutrients are the substrates for all the biochemical reactions of human metabolism and are required every day. Therefore, it is logical to assume that shortages (undernutrition) and excesses (overnutrition) have a major impact on homeostasis and consequently health outcomes or performance outcomes. The connections between athletic performance and good nutrition are well known; however, for clinical research, the question of necessity does not need to be asked (i.e., does a person who is not eating need more nutrition); rather, the focus could be on returning the individual to homeostasis (e.g., what nutrient and energy combination/ratio is required to return the individual to good nutritional status).

This paper expands our concept of utilizing nutrient ratios into the analysis of macronutrient ratios and complements our previous analysis of micronutrient ratios. A central issue is the view that macronutrients are not required in as specific quantities which may ultimately be the case, and this has resulted in a knowledge gap regarding the links between specific macronutrient intakes ratios and outcomes. Furthermore, the AMDRs may be shaping the ratio of macronutrients per meal as opposed to the overall daily intake. Differences in inter- and intra-macronutrient ratios, albeit small, also exist based on age and sex, suggesting that intake ratios change over time. There is a need to measure and report more detailed macronutrient measures as total protein, fat, and carbohydrate convey very limited information, especially when correlating these values with health outcomes. Analyzing detailed dietary macronutrient ratios may help establish better macronutrient quality benchmarks and shape future guidelines to prevent and treat modern diseases and conditions. While intra- and inter-macronutrient ratios may be applied to all types of nutritional research, nutritional epidemiology, and, ultimately, dietary guidelines, the methodology required has not been established. In the meantime, intra- and inter-macronutrient ratios may serve as a measure of individual and total macronutrient quality.

## Figures and Tables

**Table 1:** New and previous daily values (DVs) from the daily reference values for males and females aged 4 years or older.

Nutrient	New DVs [22]	Previous DVs [23]
Fat (g)	78	65
Saturated fat (g)	20	20
Carbohydrate (g)	275	300
Fiber (g)	28	25
Protein (g)	50	50
Added sugars (g)	50	--
Total macronutrient (g)	403	415
Total energy from macronutrients (kcal) *	1890	1885

* Energy was calculated using net carbohydrate (carbohydrate minus fiber × 4 kcal/g) + (total protein × 4 kcal/g) + (total fat × 9 kcal/g). Fiber was assumed to be zero calories for simplicity.

**Table 2:** The Daily Values (DVs) and food intakes (derived from the National Health and Nutrition Examination Survey (NHANES) 2007–2008 and 2015–2016 data) for males and females 51–59 years of age, their ratios, and their ratios relative to protein.

Dataset	Protein	Net CHO	Fiber	SFA	Other Fat	Row Total
DV value (g)	50	247	28	20	58	403
DV ratio	0.1241	0.0496	0.1439	0.6129	0.0695	1
DV ratio with protein set at 1	1	4.9	0.6	0.4	1.2	
Male NHANES 2007–2008 mean intake, 50–59 years (g)	100.1	262.6	18.4	32.4	67.6	481.1
Male NHANES 2007–2008 mean intake, 50–59 years ratio	0.2081	0.5458	0.0382	0.0673	0.1405	1
Male NHANES 2007–2008 mean intake, 50–59 years ratio with protein set at 1	1	2.6	0.2	0.3	0.7	
Female NHANES 2007–2008 mean intake, 50–59 years (g)	68.2	202.4	15.6	22.9	47.5	356.6
Female NHANES 2007–2008 mean intake, 50–59 years ratio	0.1913	0.5676	0.0437	0.0642	0.1332	1
Female NHANES 2007–2008 mean intake, 50–59 years ratio with protein set at 1	1	3	0.2	0.3	0.7	
Male NHANES 2015–2016 mean intake, 50–59 years (g)	97.5	265.2	18.8	32.1	66.2	479.8
Male NHANES 2015–2016 mean intake, 50–59 years ratio	0.2032	0.5527	0.0392	0.0669	0.1380	1
Male NHANES 2015–2016 mean intake, 50–59 years ratio with protein set at 1	1	2.7	0.2	0.3	0.7	
Female NHANES 2015–2016 mean intake, 50–59 years (g)	69.6	190.8	16.2	23.7	51.4	351.7
Female NHANES 2015–2016 mean intake, 50–59 years ratio	0.1979	0.5425	0.0461	0.0674	0.1461	1
Female NHANES 2015–2016 mean intake, 50–59 years ratio with protein set at 1	1	2.7	0.2	0.3	0.7	

**Table 3:** Fatty acid intakes derived from the National Health and Nutrition Examination Survey (NHANES) 2007–2008 and 2015–2016 data, their ratios, and their concentrations relative to oleic acid (n-6/n-3 polyunsaturated fatty acid (PUFA) 18:1) for males and females 51–59 years of age.

Dataset	SFA 4:0	SFA 6:0	SFA 8:0	SFA 10:0	SFA 12:0	SFA 14:0	SFA 16:0	SFA 18:0	MUFA 16:1	MUFA 18:1	MUFA 20:1	MUFA 22:1	PUFA 18:2	PUFA 18:3	PUFA 18:4	PUFA 20:4	PUFA 20:5	PUFA 22:5	PUFA 22:6
**Male NHANES 2007–2008 50–59 years of age**																			
Mean intake (g)	0.65	0.35	0.27	0.5	0.8	2.57	17.65	8.55	1.66	35.14	0.35	0.06	18.9	1.83	0.03	0.19	0.08	0.03	0.14
Intake ratio	0.0072	0.0039	0.0030	0.0056	0.0089	0.0286	0.1967	0.0953	0.0185	0.3915	0.0039	0.0007	0.2106	0.0204	0.0003	0.0021	0.0009	0.0003	0.0016
Ratio with PUFA 18:1 set at 1	0.0185	0.0100	0.0077	0.0142	0.0228	0.0731	0.5023	0.2433	0.0472	1	0.0100	0.0017	0.5378	0.0521	0.0009	0.0054	0.0023	0.0009	0.0040
**Female NHANES 2007–2008 50–59 years of age**																			
Mean intake (g)	0.51	0.27	0.24	0.41	0.8	1.94	12.08	5.89	0.99	23.73	0.21	0.04	14.38	1.38	0.01	0.11	0.05	0.02	0.09
Intake ratio	0.0081	0.0043	0.0038	0.0065	0.0127	0.0307	0.1913	0.0933	0.0157	0.3758	0.0033	0.0006	0.2277	0.0219	0.0002	0.0017	0.0008	0.0003	0.0014
Ratio with PUFA 18:1 set at 1	0.0215	0.0114	0.0101	0.0173	0.0337	0.0818	0.5091	0.2482	0.0417	1	0.0088	0.0017	0.6060	0.0582	0.0004	0.0046	0.0021	0.0008	0.0038
**Male NHANES 2015–2016 50–59 years of age**																			
Mean intake (g)	0.59	0.38	0.34	0.63	1.16	2.75	17.38	7.81	1.42	32.26	0.37	0.04	19.8	2.17	0.01	0.19	0.03	0.03	0.07
Intake ratio	0.0067	0.0043	0.0039	0.0072	0.0133	0.0315	0.1988	0.0893	0.0162	0.3690	0.0042	0.0005	0.2265	0.0248	0.0001	0.0022	0.0003	0.0003	0.0008
Ratio with PUFA 18:1 set at 1	0.0183	0.0118	0.0105	0.0195	0.0360	0.0852	0.5387	0.2421	0.0440	1	0.0115	0.0012	0.6138	0.0673	0.0003	0.0059	0.0009	0.0009	0.0022
**Female NHANES 2015–2016 50–59 years of age**																			
Mean intake (g)	0.5	0.31	0.26	0.51	0.85	2.05	12.82	5.45	0.99	24.96	0.28	0.02	15.75	1.71	0.01	0.14	0.03	0.02	0.06
Intake ratio	0.0075	0.0046	0.0039	0.0076	0.0127	0.0307	0.1921	0.0817	0.0148	0.3741	0.0042	0.0003	0.2361	0.0256	0.0001	0.0021	0.0004	0.0003	0.0009
Ratio with PUFA 18:1 set at 1	0.0200	0.0124	0.0104	0.0204	0.0341	0.0821	0.5136	0.2183	0.0397	1	0.0112	0.0008	0.6310	0.0685	0.0004	0.0056	0.0012	0.0008	0.0024

Abbreviations: butyric acid (SFA 4:0), caproic acid (SFA 6:0), caprylic acid (SFA 8:0), capric acid (SFA 10:0), lauric acid (SFA 12:0), myristic acid (SFA 14:0), palmitic acid (SFA 16:0), stearic acid (SFA 18:0), palmitoleic acid *n-7* (MUFA 16:1), oleic acid *n-9* (MUFA 18:1), gondoic acid *n-9* (MUFA 20:1), erucic acid *n-9* (MUFA 22:1), linoleic acid *n-6* (PUFA 18:2), alpha-linolenic acid *n-3* (PUFA 18:3), stearidonic acid *n-3* (PUFA 18:4), arachadonic acid *n-6* (PUFA 20:4), eicosapentaonic acid *n-3* (PUFA 20:5), docosapentaenoic acid *n-3* (PUFA 22:5), and docosahexaenoic acid *n-3* (PUFA 22:6).

**Table 4:** Macronutrient intakes (derived from the National Health and Nutrition Examination Survey (NHANES) 2007–2008 and 2015–2016 data) their ratios, and their ratios relative to protein at meal occasions for males and females 51–59 years of age.

Dataset	Protein	Net CHO	Fiber	SFA	Other Fat
Breakfast					
Male NHANES 2007–2008 mean intake	14.01	46.90	3.68	5.18	10.82
Male NHANES 2007–2008 mean intake, 50–59 years ratio	0.17	0.58	0.05	0.06	0.13
Male NHANES 2007–2008 mean intake, 50–59 years ratio with protein set at 1	1	3.35	0.26	0.37	0.77
Female NHANES 2007–2008 mean intake	10.23	38.46	2.96	3.21	6.65
Female NHANES 2007–2008 mean intake, 50–59 years ratio	0.17	0.63	0.05	0.05	0.11
Female NHANES 2007–2008 mean intake, 50–59 years ratio with protein set at 1	1	3.76	0.29	0.31	0.65
Male NHANES 2015–2016 mean intake	16.58	50.39	3.57	5.78	9.95
Male NHANES 2015–2016 mean intake, 50–59 years ratio	0.19	0.58	0.04	0.07	0.12
Male NHANES 2015–2016 mean intake, 50–59 years ratio with protein set at 1	1	3.04	0.22	0.35	0.60
Female NHANES 2015–2016 mean intake	12.53	38.00	3.40	4.27	8.50
Female NHANES 2015–2016 mean intake, 50–59 years ratio	0.19	0.57	0.05	0.06	0.13
Female NHANES 2015–2016 mean intake, 50–59 years ratio with protein set at 1	1	3.03	0.27	0.34	0.68
Lunch					
Male NHANES 2007–2008 mean intake	28.03	57.04	4.78	8.42	18.58
Male NHANES 2007–2008 mean intake, 50–59 years ratio	0.24	0.49	0.04	0.07	0.16
Male NHANES 2007–2008 mean intake, 50–59 years ratio with protein set at 1	1	2.03	0.17	0.30	0.66
Female NHANES 2007–2008 mean intake	18.41	44.37	3.59	5.50	12.81
Female NHANES 2007–2008 mean intake, 50–59 years ratio	0.22	0.52	0.04	0.06	0.15
Female NHANES 2007–2008 mean intake, 50–59 years ratio with protein set at 1	1	2.41	0.19	0.30	0.70
Male NHANES 2015–2016 mean intake	28.28	57.59	4.89	8.03	18.52
Male NHANES 2015–2016 mean intake, 50–59 years ratio	0.24	0.49	0.04	0.07	0.16
Male NHANES 2015–2016 mean intake, 50–59 years ratio with protein set at 1	1	2.04	0.17	0.28	0.65
Female NHANES 2015–2016 mean intake	16.70	41.81	3.73	4.98	11.55
Female NHANES 2015–2016 mean intake, 50–59 years ratio	0.21	0.53	0.05	0.06	0.15
Female NHANES 2015–2016 mean intake, 50–59 years ratio with protein set at 1	1	2.50	0.22	0.30	0.69
Dinner					
Male NHANES 2007–2008 mean intake	46.05	83.30	6.62	12.31	25.69
Male NHANES 2007–2008 mean intake, 50–59 years ratio	0.26	0.48	0.04	0.07	0.15
Male NHANES 2007–2008 mean intake, 50–59 years ratio with protein set at 1	1	1.81	0.14	0.27	0.56
Female NHANES 2007–2008 mean intake	30.69	63.83	5.93	8.70	18.05
Female NHANES 2007–2008 mean intake, 50–59 years ratio	0.24	0.50	0.05	0.07	0.14
Female NHANES 2007–2008 mean intake, 50–59 years ratio with protein set at 1	1	2.08	0.19	0.28	0.59
Male NHANES 2015–2016 mean intake	40.95	83.74	7.14	12.20	26.14
Male NHANES 2015–2016 mean intake, 50–59 years ratio	0.24	0.49	0.04	0.07	0.15
Male NHANES 2015–2016 mean intake, 50–59 years ratio with protein set at 1	1	2.04	0.17	0.30	0.64
Female NHANES 2015–2016 mean intake	31.32	60.25	5.99	9.48	20.56
Female NHANES 2015–2016 mean intake, 50–59 years ratio	0.25	0.47	0.05	0.07	0.16
Female NHANES 2015–2016 mean intake, 50–59 years ratio with protein set at 1	1	1.92	0.19	0.30	0.66
All snacks					
Male NHANES 2007–2008 mean intake	12.01	72.56	3.31	6.16	11.84
Male NHANES 2007–2008 mean intake, 50–59 years ratio	0.11	0.69	0.03	0.06	0.11
Male NHANES 2007–2008 mean intake, 50–59 years ratio with protein set at 1	1	6.04	0.28	0.51	0.99
Female NHANES 2007–2008 mean intake	8.87	55.74	3.12	5.50	9.99
Female NHANES 2007–2008 mean intake, 50–59 years ratio	0.11	0.67	0.04	0.07	0.12
Female NHANES 2007–2008 mean intake, 50–59 years ratio with protein set at 1	1	6.29	0.35	0.62	1.13
Male NHANES 2015–2016 mean intake	11.70	76.32	3.20	6.10	10.61
Male NHANES 2015–2016 mean intake, 50–59 years ratio	0.11	0.71	0.03	0.06	0.10
Male NHANES 2015–2016 mean intake, 50–59 years ratio with protein set at 1	1	6.52	0.27	0.52	0.91
Female NHANES 2015–2016 mean intake	9.05	50.90	2.92	4.98	10.79
Female NHANES 2015–2016 mean intake, 50–59 years ratio	0.12	0.65	0.04	0.06	0.14
Female NHANES 2015–2016 mean intake, 50–59 years ratio with protein set at 1	1	5.63	0.32	0.55	1.19

Values are rounded.

**Table 5:** The mean NHANES intake (derived from the National Health and Nutrition Examination Survey (NHANES) 2007–2008 and 2015–2016 data) of sugars, fiber, and the remaining carbohydrates, their ratios, and their ratios relative to fiber for males and females 51–59 years of age.

Dataset	Sugars	Fiber	Remaining CHO
Male 50–59 years NHANES 2007–2008			
Mean intake (g)	123	18.4	139.6
Intake as percent of total CHO (%)	43.77	6.55	49.68
Ratio	0.43772	0.06548	0.49680
Ratio with fiber set at 1	6.68	1	7.59
Female 50–59 years NHANES 2007–2008			
Mean intake (g)	102	15.6	100.4
Intake as percent of total CHO (%)	46.79	7.16	46.06
Ratio	0.46789	0.07156	0.46055
Ratio with fiber set at 1	6.54	1	6.44
Male 50–59 years NHANES 2015–2016			
Mean intake (g)	125	18.8	140.2
Intake as percent of total CHO (%)	44.01	6.62	49.37
Ratio	0.44014	0.06620	0.49366
Ratio with fiber set at 1	6.65	1	7.46
Female 50–59 years NHANES 2015–2016			
Mean intake (g)	93	16.2	97.8
Intake as percent of total CHO (%)	44.93	7.83	47.25
Ratio	0.44928	0.07826	0.47246
Ratio with fiber set at 1	5.74	1	6.04

Remaining CHO = total carbohydrate − (sugars + fiber).

**Table 6:** Macronutrient Daily Values (DV) for males and females 51–70 years of age, the percentage contribution from each macronutrient to the total, the intakes from the National Health and Nutrition Examination Survey (NHANES) 2015–2016 for males and females aged 51–59 years, and the projected intake, per DV proportions, based on the total intake.

Nutrient	DV (g)	DV % of Total (%)	% Total Energy	Male				Female			
				NHANES Data, Age 50–59 (g)	% Total Energy	NHANES in Proportion to DV (g)	% Total Energy	NHANES Data, Age 50–59 (g)	% Total Energy	NHANES in Proportion to DV (g)	% Total Energy
**2007–2008**											
**Protein**	50	12.4	10.6	100.1	17.0	59.7	10.6	68.2	15.9	44.2	10.6
**Net CHO**	247	61.3	52.3	262.6	44.7	294.9	52.3	202.4	47.2	218.6	52.3
Fiber *	28	6.9	0.0	18.4	0.0	33.4	0.0	15.6	0.0	24.8	0.0
**SFA**	20	5.0	9.5	32.4	12.4	23.9	9.5	22.9	12.0	17.7	9.5
**Other fat**	58	14.4	27.6	67.6	25.9	69.2	27.6	47.5	24.9	51.3	27.6
**TOTAL**	403	100		481.1		481.1		356.6		356.6	
**2015–2016**											
**Protein**	50	12.4	10.6	97.5	16.7	59.5	10.6	69.6	16.2	43.6	10.6
**Net CHO**	247	61.3	52.3	265.2	45.4	294.1	52.3	190.8	44.4	215.6	52.3
Fiber *	28	6.9	0.0	18.8	0.0	33.3	0.0	16.2	0.0	24.4	0.0
**SFA**	20	5.0	9.5	32.1	12.4	23.8	9.5	23.7	12.4	17.5	9.5
**Other fat**	58	14.4	27.6	66.2	25.5	69.1	27.6	51.4	26.9	50.6	27.6
**TOTAL**	403	100		479.8		479.8		351.7		351.7	

* Fiber was assumed to be zero calories for simplicity.

## Supplementary Materials

The following are available online at http://www.mdpi.com/2072-6643/11/2/282/s1, Figure S1: Total macronutrient intakes at breakfast, lunch, and dinner and for all snacks combined for males and females of 51–59 years of age, derived from the National Health and Nutrition Examination Survey (NHANES) 2007–2008 and 2015–2016 data, Figure S2: The total protein intakes at breakfast, lunch, and dinner and for all snacks combined for males and females of 51–59 years of age, derived from the National Health and Nutrition Examination Survey (NHANES) 2007–2008 and 2015–2016 data, Figure S3: The total net carbohydrate (Net CHO) intakes at breakfast, lunch, and dinner and for all snacks combined for males and females of 51–59 years of age, derived from the National Health and Nutrition Examination Survey (NHANES) 2007–2008 and 2015–2016 data, Figure S4: The total fiber intakes at breakfast, lunch, and dinner and for all snacks combined for males and females of 51–59 years of age, derived from the National Health and Nutrition Examination Survey (NHANES) 2007–2008 and 2015–2016 data, Figure S5: The total saturated fat (SFA) intakes at breakfast, lunch, and dinner and for all snacks combined for males and females of 51–59 years, derived from the National Health and Nutrition Examination Survey (NHANES) 2007–2008 and 2015–2016 data, Figure S6: The total Other fat intakes at breakfast, lunch, and dinner and for all snacks combined for males and females of 51–59 years of age, derived from the National Health and Nutrition Examination Survey (NHANES) 2007–2008 and 2015–2016 data, Figure S7: Individual fatty acid intake values (derived from the National Health and Nutrition Examination Survey (NHANES) 2007–2008 and 2015–2016 data) for males and females of 50–59 years of age, Figure S8. Male fatty acid intake ratios, as a percent of total, for all fatty acids, saturated fatty acids, monounsaturated fatty acids, and polyunsaturated fatty acids, derived from the National Health and Nutrition Examination Survey (NHANES) 2007–2008 and 2015–2016 data for males and females of 51–59 years of age, Figure S9. Female fatty acid intake ratios, as a percent of total, for all fatty acids, saturated fatty acids, monounsaturated fatty acids, and polyunsaturated fatty acids, derived from the National Health and Nutrition Examination Survey (NHANES) 2007–2008 and 2015–2016 data for males and females of 51–59 years, Figure S10. Mean daily male individual and total macronutrient intakes derived from the National Health and Nutrition Examination Survey (NHANES) 2001–2016 data, across various age groups, as a percentage of the daily value (DV; 100% = daily value), Figure S11: Mean daily female individual and total macronutrient intakes derived from the National Health and Nutrition Examination Survey (NHANES) 2001–2016 data, across various age groups, as a percentage of the daily value (DV; 100% = daily value).
