# Supplementary material for: Utilizing Dietary Nutrient Ratios in Nutritional Research: Expanding the Concept of Nutrient Ratios to Macronutrients
Source: Nutrients. 2019 Jan 28;11(2):282. doi: 10.3390/nu11020282 (PMC6413020; doi:10.3390/nu11020282)
Supplement: Supplementary file 1 [file nutrients-11-00282-s001.zip › Supplemental materials/Suppl. figure legends.docx]

**Supplemental Figure 1. Total macronutrient intakes at breakfast, lunch, dinner and for all snacks combined, for males and females 51-59 years, derived from National Health and Nutrition Examination Survey (NHANES) 2007-2008 and 2015-2016 data.**

**Supplemental Figure 2. The total protein intakes at breakfast, lunch, dinner and for all snacks combined, for males and females 51-59 years, derived from National Health and Nutrition Examination Survey (NHANES) 2007-2008 and 2015-2016 data.**

**Supplemental Figure 3. The total net carbohydrate (Net CHO) intakes at breakfast, lunch, dinner and for all snacks combined, for males and females 51-59 years, derived from National Health and Nutrition Examination Survey (NHANES) 2007-2008 and 2015-2016 data.**

**Supplemental Figure 4. The total fiber intakes at breakfast, lunch, dinner and for all snacks combined, for males and females 51-59 years, derived from National Health and Nutrition Examination Survey (NHANES) 2007-2008 and 2015-2016 data.**

**Supplemental Figure 5. The total saturated fat (SFA) intakes at breakfast, lunch, dinner and for all snacks combined, for males and females 51-59 years, derived from National Health and Nutrition Examination Survey (NHANES) 2007-2008 and 2015-2016 data.**

**Supplemental Figure 6. The total Other fat intakes at breakfast, lunch, dinner and for all snacks combined, for males and females 51-59 years, derived from National Health and Nutrition Examination Survey (NHANES) 2007-2008 and 2015-2016 data.**

**Supplemental Figure 7. Individual fatty acid intake values (derived from National Health and Nutrition Examination Survey (NHANES) 2007-2008 and 2015-2016 data) for males and females 50-59 years. Y axes are log_10_ scale.**

**Supplemental Figure 8. Male fatty acid intake ratios, as a percent of total, for all fatty acids, saturated fatty acids, monounsaturated fatty acids and polyunsaturated fatty acids, derived from National Health and Nutrition Examination Survey (NHANES) 2007-2008 and 2015-2016 data, for males and females 51-59 years.**

Data are expressed as the percent of total intake. The secondary pie chart contains lower values.

Abbreviations: Butyric acid (SFA 4:0), Caproic acid (SFA 6:0), Caprylic acid (SFA 8:0), Capric acid (SFA 10:0), Lauric acid (SFA 12:0), Myristic acid (SFA 14:0), Palmitic acid (SFA 16:0), Stearic acid (SFA 18:0), Palmitoleic acid *n-7* (MUFA 16:1), Oleic acid *n-9* (MUFA 18:1), Gondoic acid *n-9* (MUFA 20:1), Erucic acid *n-9* (MUFA 22:1), Linoleic acid *n-6* (PUFA 18:2), Alpha-linolenic acid *n-3* (PUFA 18:3), Stearidonic acid *n-3* (PUFA 18:4), Arachadonic acid *n-6* (PUFA 20:4), Eicosapentaonic acid *n-3* (PUFA 20:5), Docosapentaenoic acid *n-3* (PUFA 22:5), Docosahexanoic acid *n-3* (PUFA 22:6)

**Supplemental Figure 9. Female fatty acid intake ratios, as a percent of total, for all fatty acids, saturated fatty acids, monounsaturated fatty acids and polyunsaturated fatty acids, derived from National Health and Nutrition Examination Survey (NHANES) 2007-2008 and 2015-2016 data, for males and females 51-59 years.**

Data are expressed as the percent of total intake. The secondary pie chart contains lower values.

Abbreviations: Butyric acid (SFA 4:0), Caproic acid (SFA 6:0), Caprylic acid (SFA 8:0), Capric acid (SFA 10:0), Lauric acid (SFA 12:0), Myristic acid (SFA 14:0), Palmitic acid (SFA 16:0), Stearic acid (SFA 18:0), Palmitoleic acid *n-7* (MUFA 16:1), Oleic acid *n-9* (MUFA 18:1), Gondoic acid *n-9* (MUFA 20:1), Erucic acid *n-9* (MUFA 22:1), Linoleic acid *n-6* (PUFA 18:2), Alpha-linolenic acid *n-3* (PUFA 18:3), Stearidonic acid *n-3* (PUFA 18:4), Arachadonic acid *n-6* (PUFA 20:4), Eicosapentaonic acid *n-3* (PUFA 20:5), Docosapentaenoic acid *n-3* (PUFA 22:5), Docosahexanoic acid *n-3* (PUFA 22:6)

**Supplemental Figure 10. Mean daily male individual and total macronutrient intakes derived from National Health and Nutrition Examination Survey (NHANES) 2001-2016 data, across various age groups, as a percentage of the Daily Value (DV; 100% = Daily Value).**

**Supplemental Figure 11. Mean daily female individual and total macronutrient intakes derived from National Health and Nutrition Examination Survey (NHANES) 2001-2016 data, across various age groups, as a percentage of the Daily Value (DV; 100% = Daily Value).**
