# Supplementary figures and images for: Utilizing Dietary Nutrient Ratios in Nutritional Research: Expanding the Concept of Nutrient Ratios to Macronutrients
Source: Nutrients. 2019 Jan 28;11(2):282. doi: 10.3390/nu11020282 (PMC6413020; doi:10.3390/nu11020282)

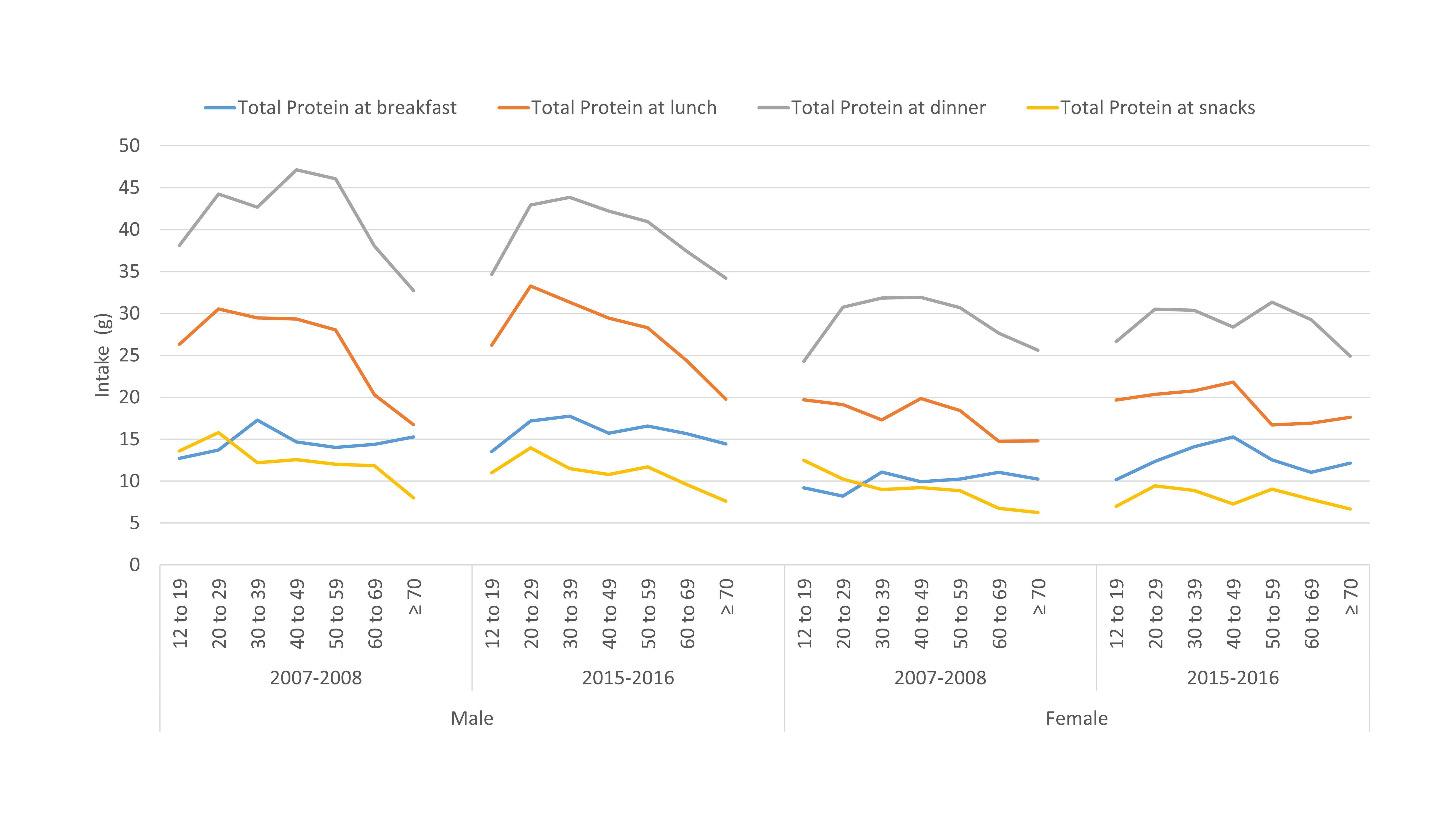

Supplement: Supplementary file 1 [file nutrients-11-00282-s001.zip › Supplemental materials/Kelly_Suppl figure 1.TIF]

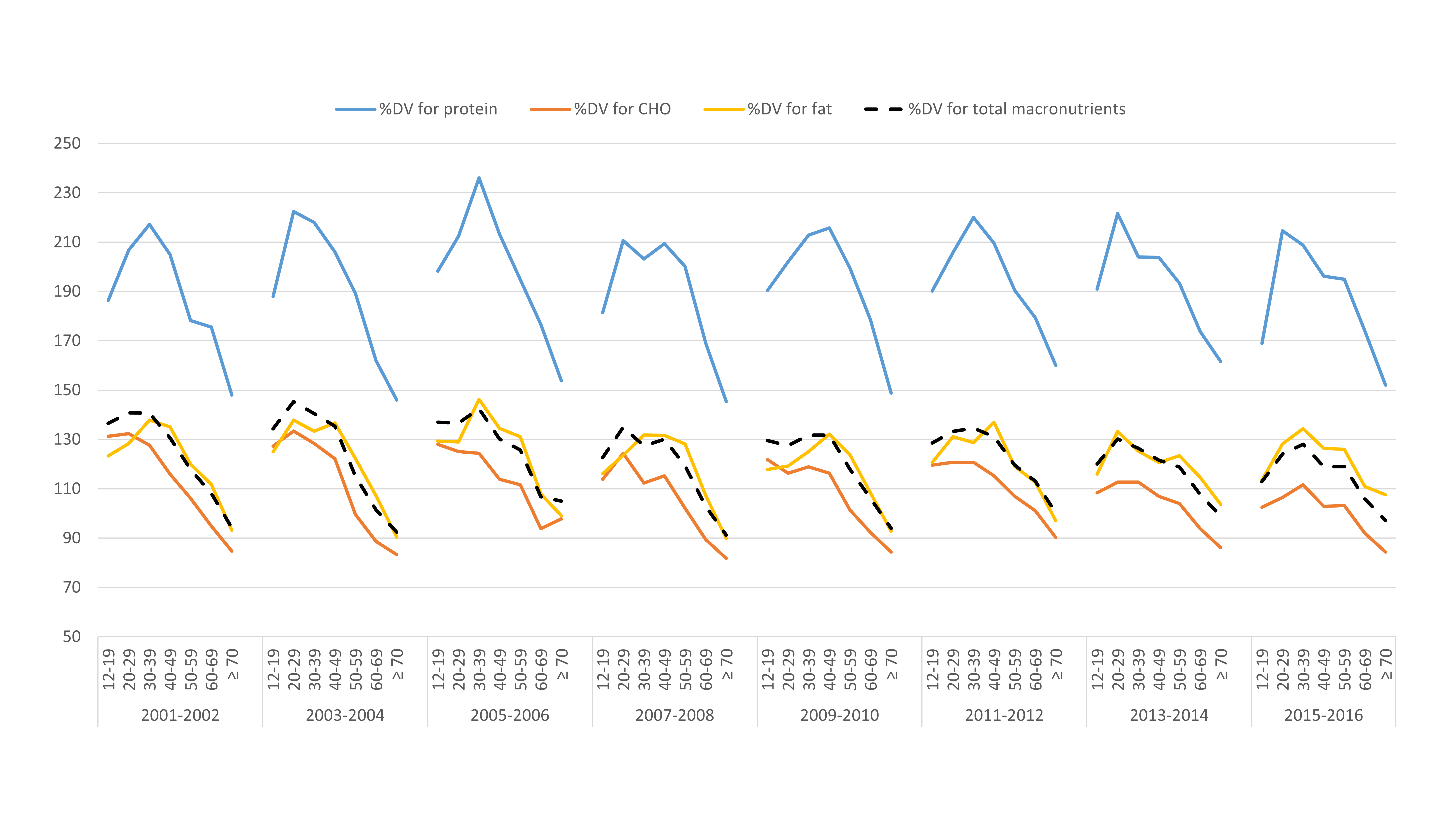

Supplement: Supplementary file 1 [file nutrients-11-00282-s001.zip › Supplemental materials/Kelly_Suppl figure 10.TIF]

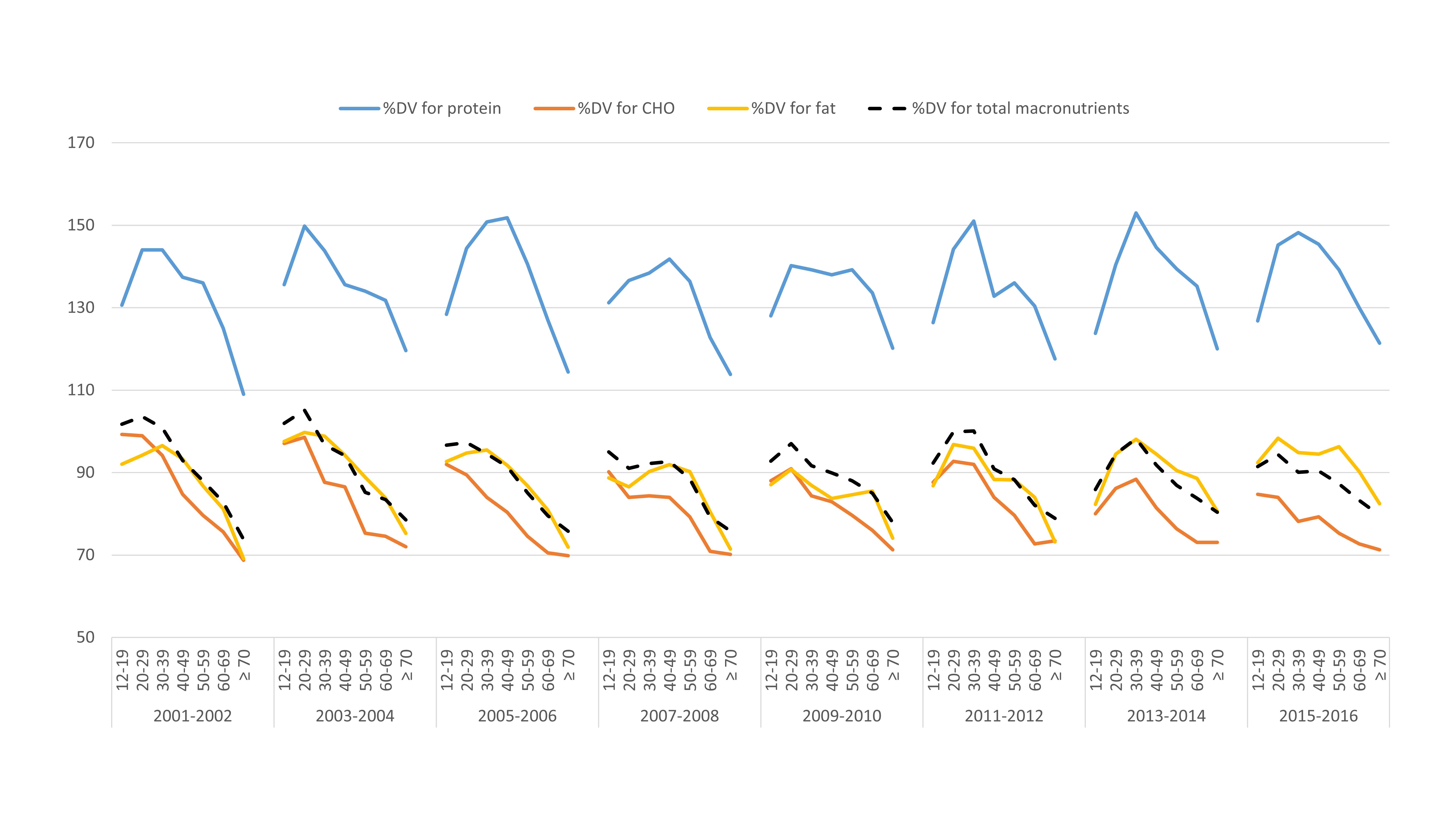

Supplement: Supplementary file 1 [file nutrients-11-00282-s001.zip › Supplemental materials/Kelly_Suppl figure 11.TIF]

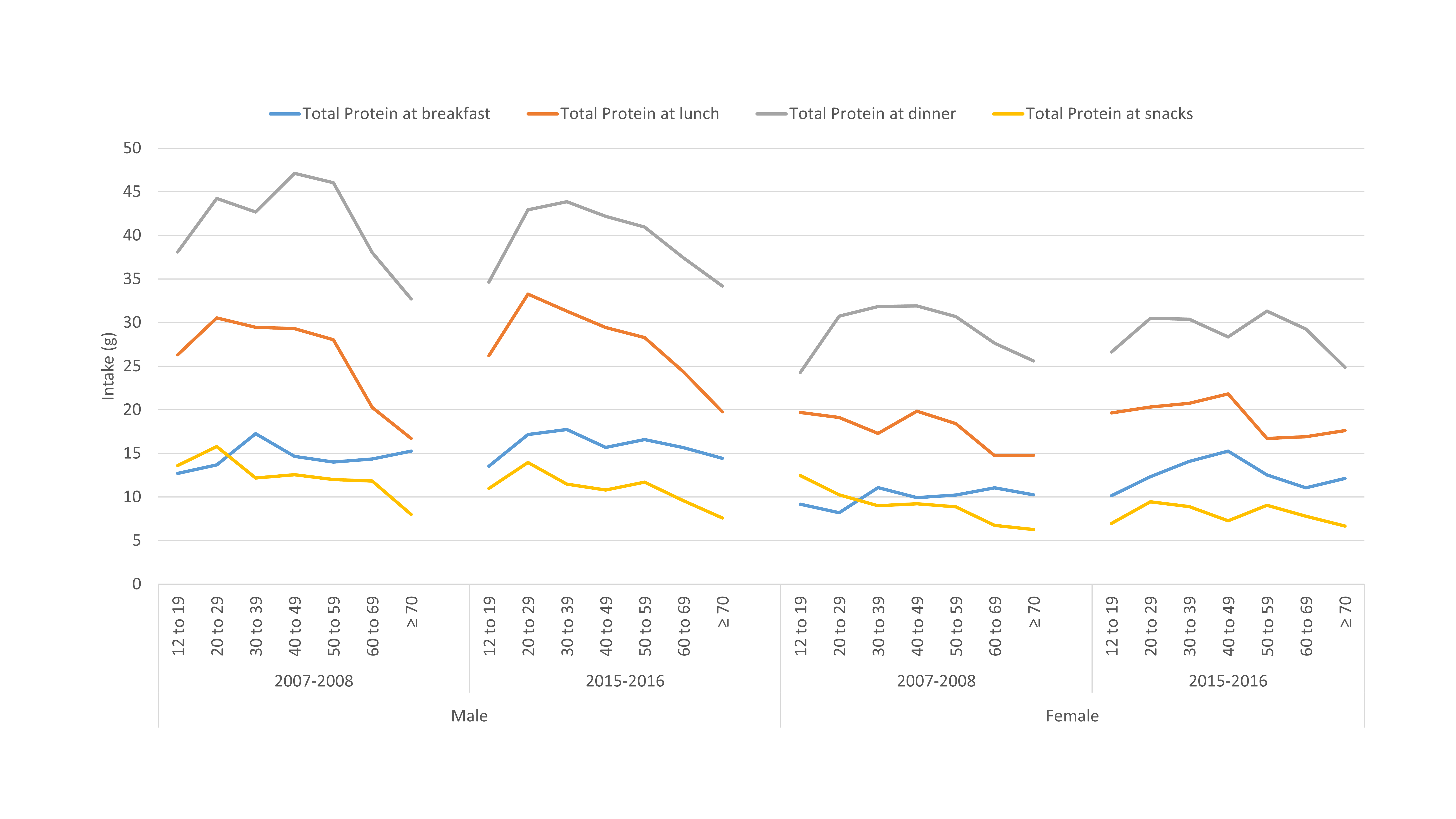

Supplement: Supplementary file 1 [file nutrients-11-00282-s001.zip › Supplemental materials/Kelly_Suppl figure 2.TIF]

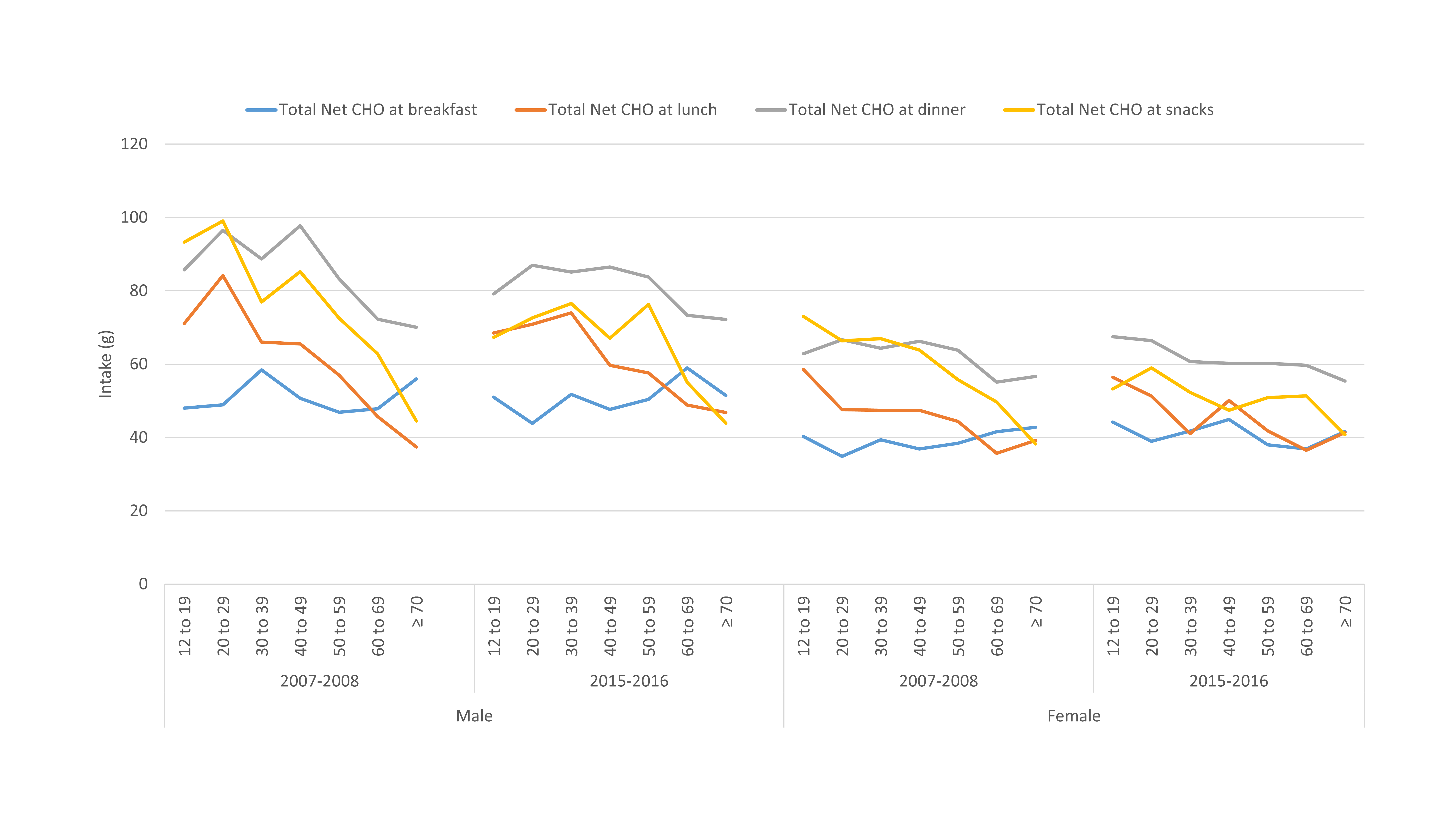

Supplement: Supplementary file 1 [file nutrients-11-00282-s001.zip › Supplemental materials/Kelly_Suppl figure 3.TIF]

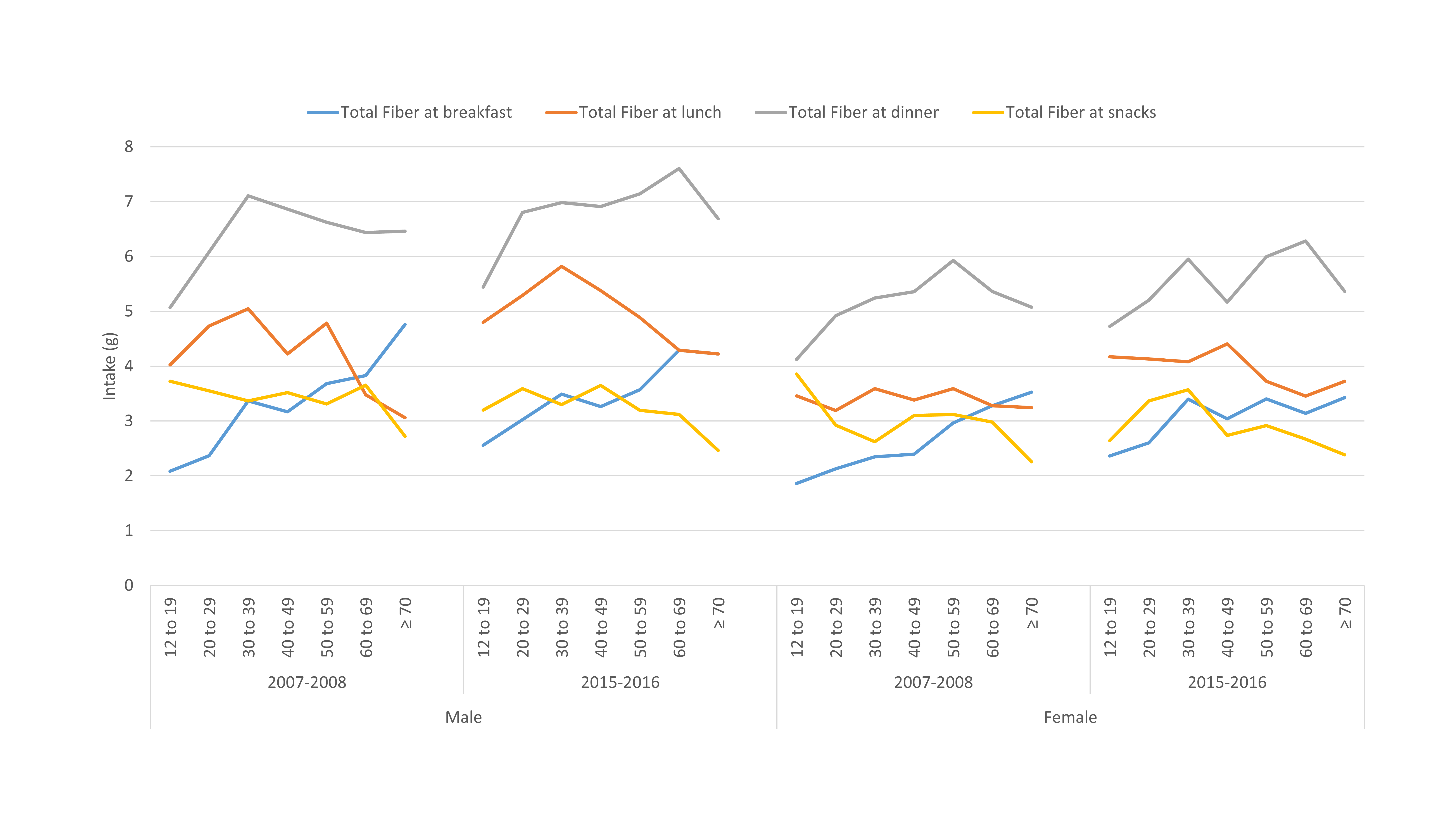

Supplement: Supplementary file 1 [file nutrients-11-00282-s001.zip › Supplemental materials/Kelly_Suppl figure 4.TIF]

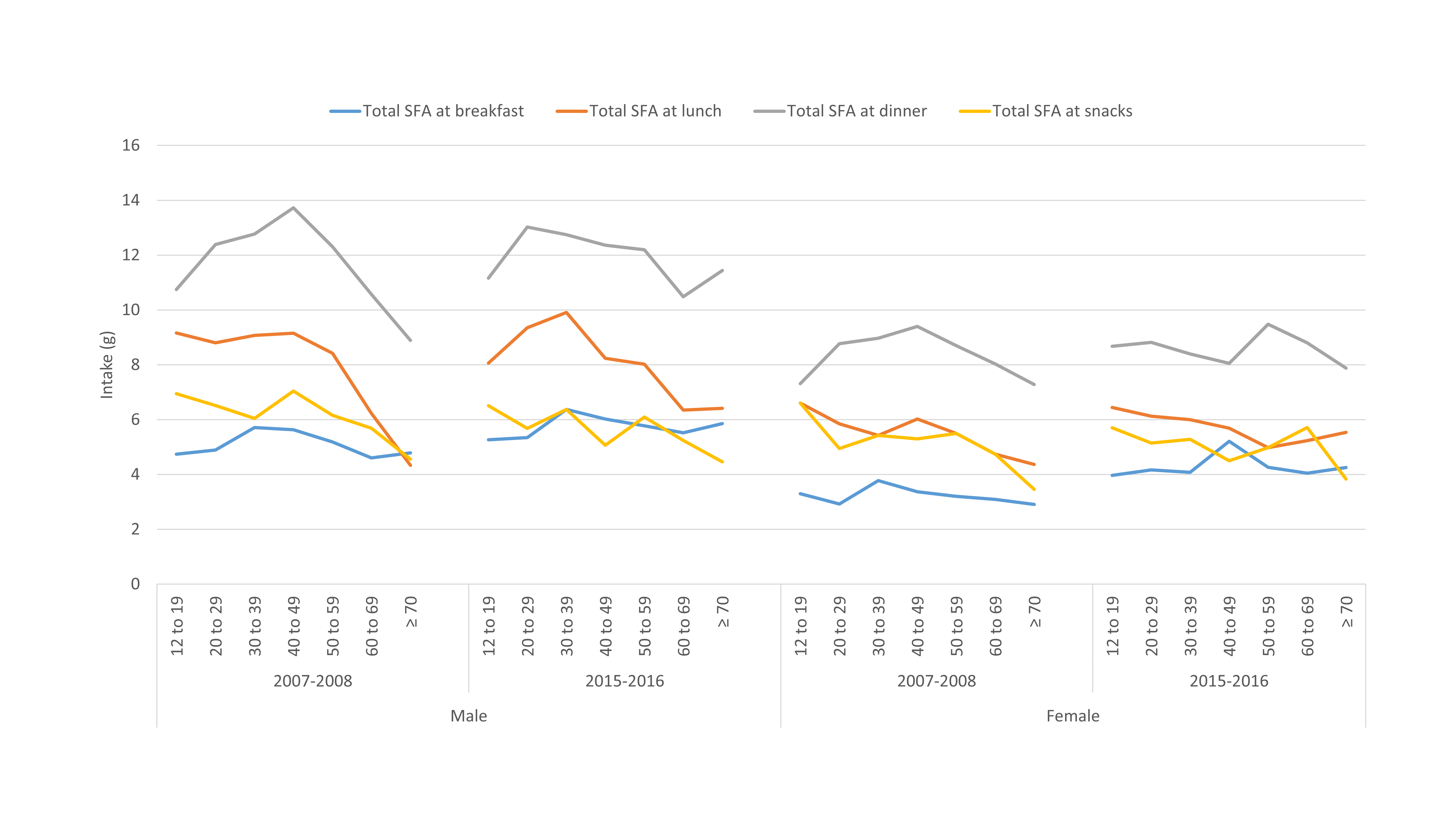

Supplement: Supplementary file 1 [file nutrients-11-00282-s001.zip › Supplemental materials/Kelly_Suppl figure 5.TIF]

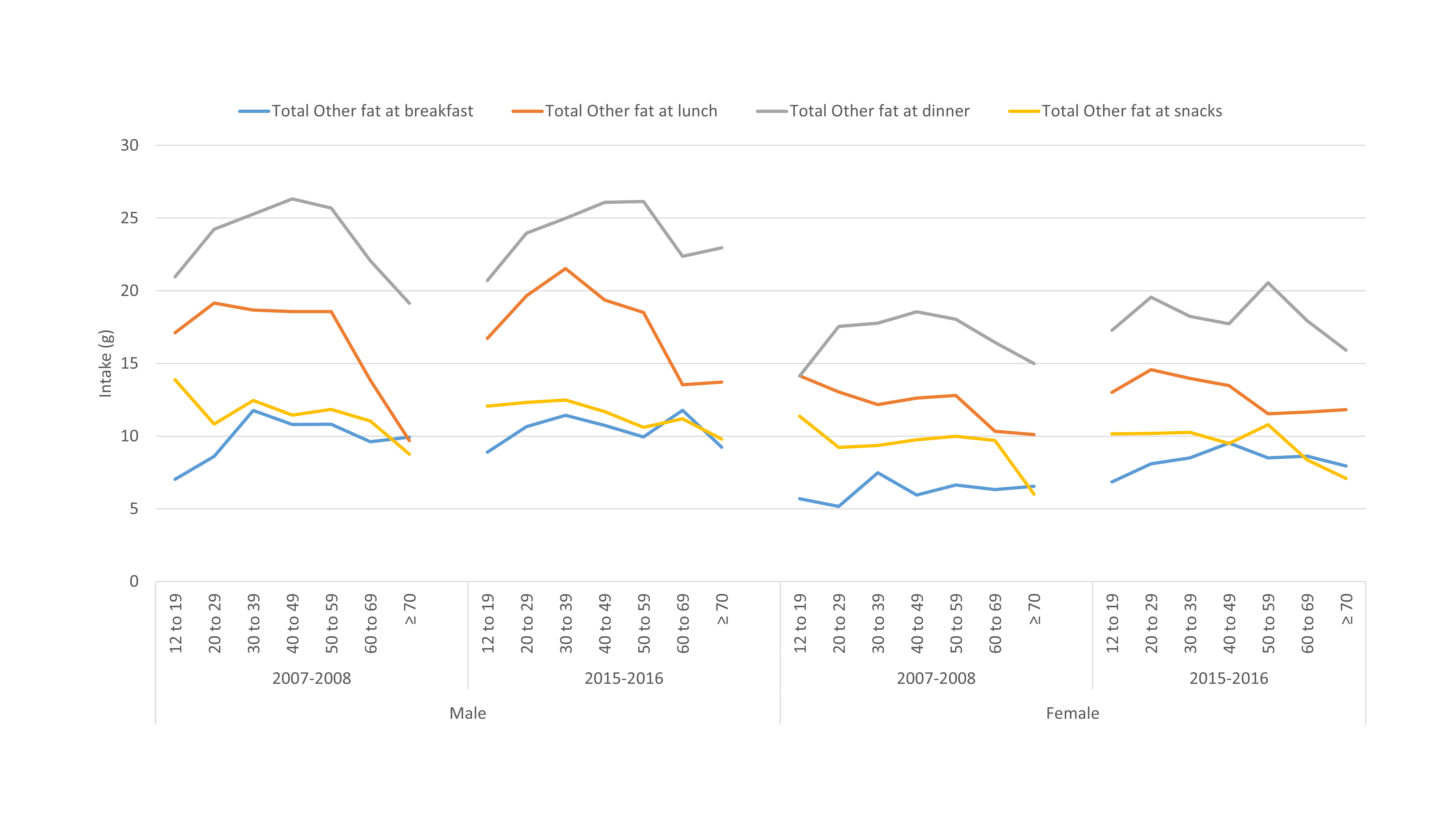

Supplement: Supplementary file 1 [file nutrients-11-00282-s001.zip › Supplemental materials/Kelly_Suppl figure 6.TIF]

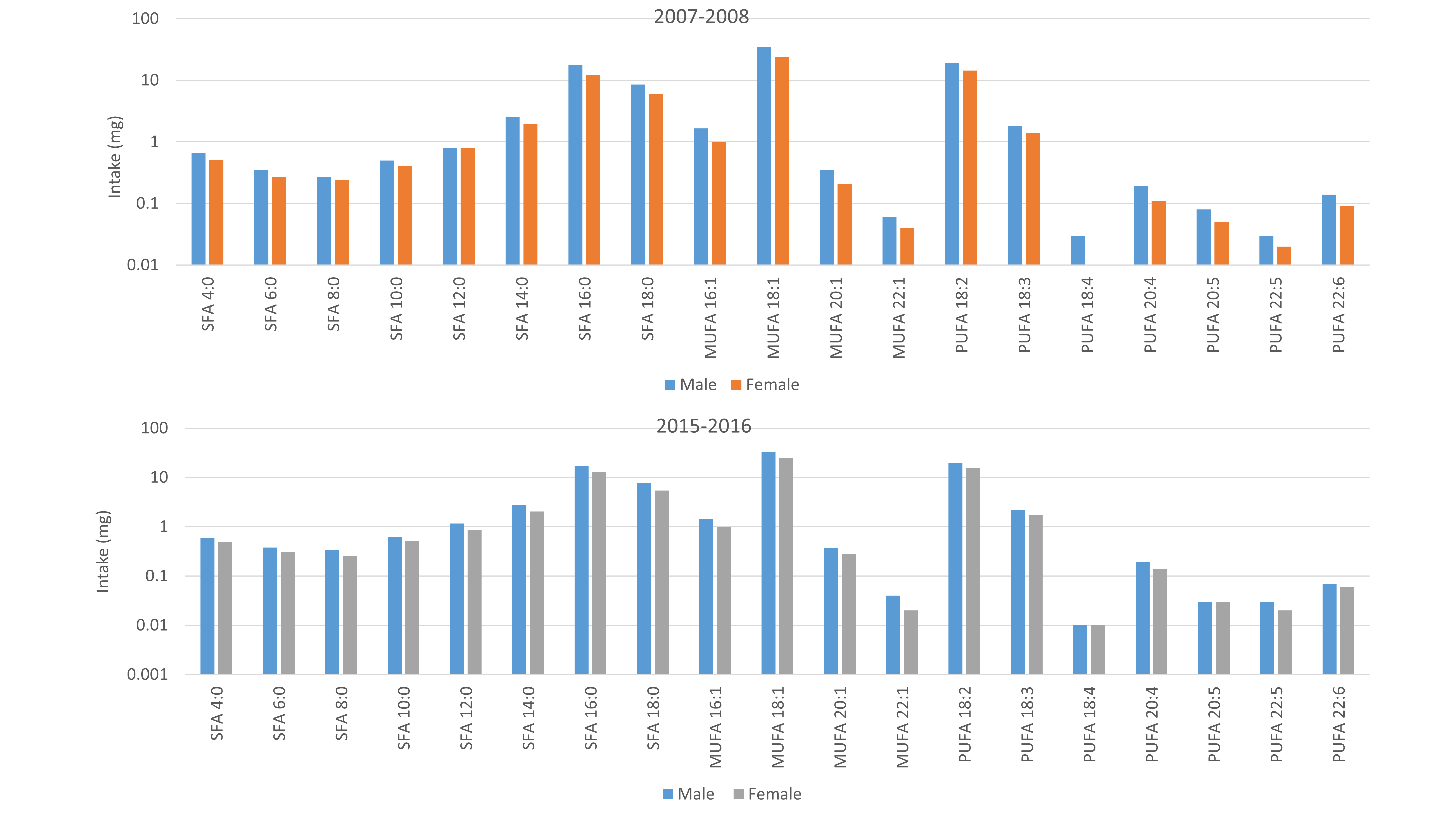

Supplement: Supplementary file 1 [file nutrients-11-00282-s001.zip › Supplemental materials/Kelly_Suppl figure 7.TIF]

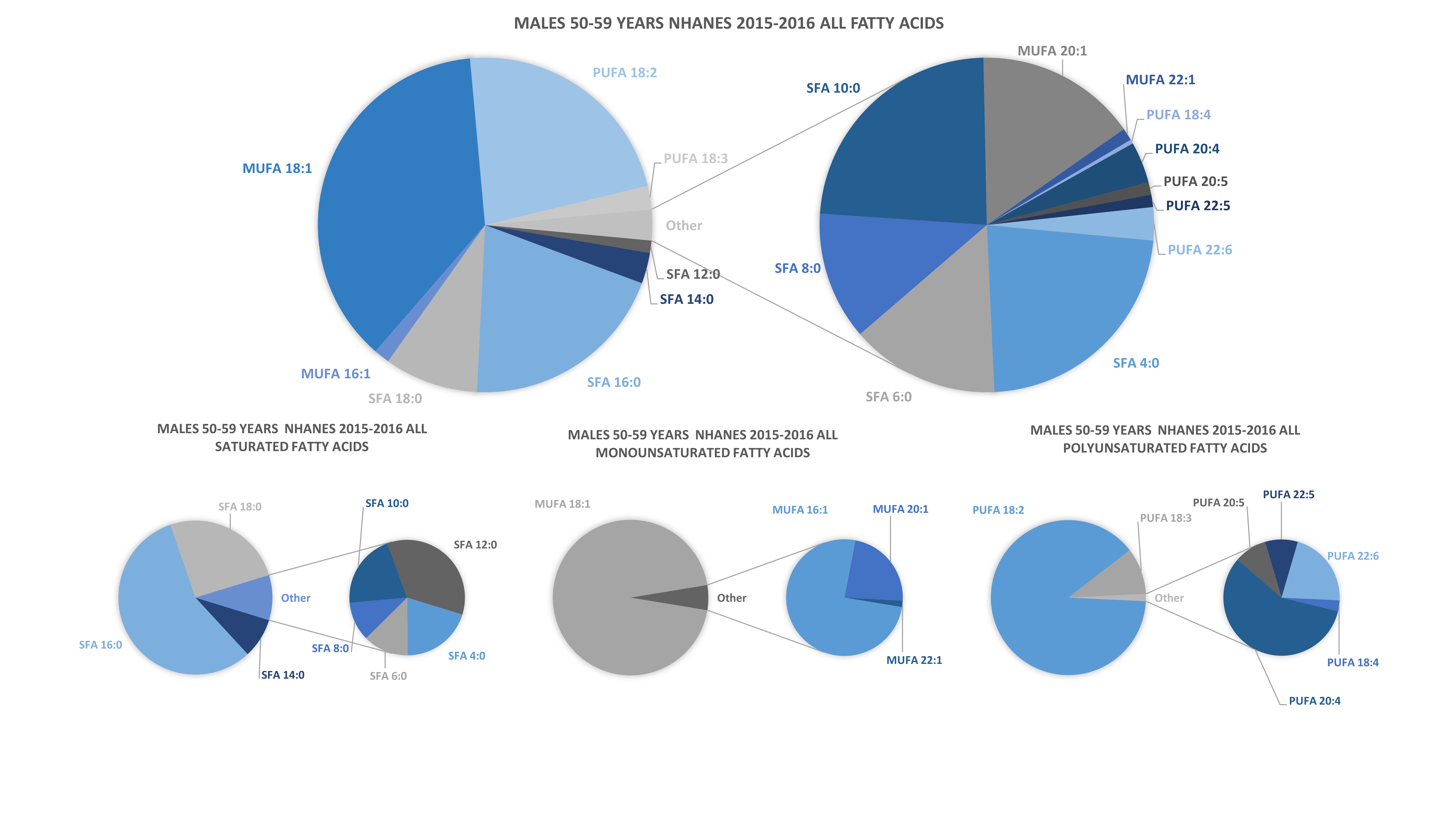

Supplement: Supplementary file 1 [file nutrients-11-00282-s001.zip › Supplemental materials/Kelly_Suppl figure 8.TIF]

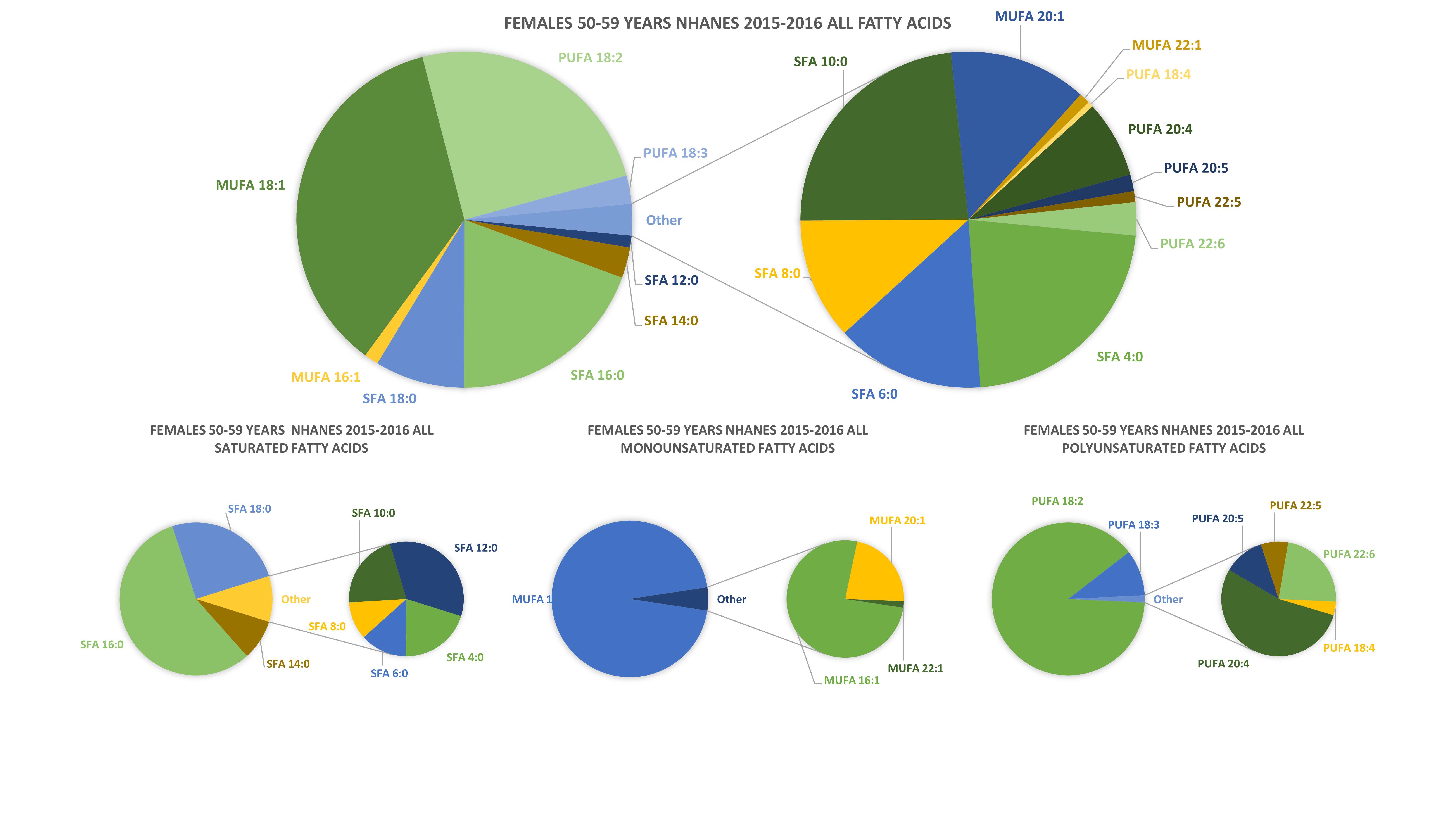

Supplement: Supplementary file 1 [file nutrients-11-00282-s001.zip › Supplemental materials/Kelly_Suppl figure 9.TIF]
